# Lifelong smoking trajectories of Northern Finns are characterized by sociodemographic and lifestyle differences in a 46-year follow-up

**DOI:** 10.1038/s41598-020-73334-3

**Published:** 2020-10-01

**Authors:** Petteri Oura, Ina Rissanen, Juho-Antti Junno, Terttu Harju, Markus Paananen

**Affiliations:** 1 Center for Life Course Health Research, Faculty of Medicine, University of Oulu, PO Box 5000, 90014 Oulu, Finland; 2 Kerava Health Care Center, Metsolantie 2, 04200 Kerava, Finland; 3 Departments of Neurology and Neurosurgery, Oulu University Hospital, PO Box 50, 90029 Oulu, Finland; 4 Medical Research Center Oulu (MRC Oulu), Northern Ostrobothnia Hospital District, University of Oulu, PO Box 5000, 90014 Oulu, Finland; 5 Julius Center for Health Sciences and Primary Care, University Medical Center Utrecht, PO Box 85500, 3508 GA Utrecht, The Netherlands; 6 Cancer Research and Translational Medicine Research Unit, University of Oulu, PO Box 5000, 90014 Oulu, Finland; 7 Research Unit of Internal Medicine, Faculty of Medicine, University of Oulu, PO Box 5000, 90014 Oulu, Finland

**Keywords:** Medical research, Risk factors

## Abstract

Smoking remains among the leading causes of mortality worldwide. Obtaining a comprehensive understanding of a population’s smoking behaviour is essential for tobacco control. Here, we aim to characterize lifelong smoking patterns and explore underlying sociodemographic and lifestyle factors in a population-based birth cohort population followed up for 46 years. Our analysis is based on 5797 individuals from the Northern Finland Birth Cohort 1966 who self-reported their tobacco smoking behaviour at the ages of 14, 31 and 46. Data on sex, education, employment, body mass index, physical activity, alcohol consumption, and substance addiction were also collected at the follow-ups. We profile each individual’s annual smoking history from the age of 5 to 47, and conduct a latent class trajectory analysis on the data. We then characterize the identified smoking trajectory classes in terms of the background variables, and compare the heaviest smokers with other classes in order to reveal specific predictors of non-smoking and discontinued smoking. Six smoking trajectories are identified in our sample: never-smokers (class size 41.0%), youth smokers (12.6%), young adult quitters (10.8%), late adult quitters (10.5%), late starters (4.3%), and lifetime smokers (20.7%). Smoking is generally associated with male sex, lower socioeconomic status and unhealthier lifestyle. Multivariable between-class comparisons identify unemployment (odds ratio [OR] 1.28–1.45) and physical inactivity (OR 1.20–1.52) as significant predictors of lifetime smoking relative to any other class. Female sex increases the odds of never-smoking and youth smoking (OR 1.29–1.33), and male sex increases the odds of adult quitting (OR 1.30–1.41), relative to lifetime smoking. We expect future initiatives to benefit from our data by exploiting the identified predictors as direct targets of intervention, or as a means of identifying individuals who may benefit from such interventions.

## Introduction

By damaging general health and increasing the risk of several chronic diseases, smoking remains among the leading causes of mortality worldwide^1–4^. Despite the progress in smoking reduction made by the World Health Organization’s Framework Convention on Tobacco Control (WHO FCTC)^3,5^, a recent worldwide analysis predicted that over one billion individuals will remain mokers in 2025 if current smoking trends remain constant^6^. In order to reduce smoking effectively, tobacco control policies and cessation support should be based on a comprehensive characterization of a population’s smoking behaviour and the associated underlying factors. For example, the WHO FCTC includes a requirement for the ‘surveillance of the magnitude, patterns, determinants and consequences of tobacco consumption’^5^, and in the US, the identification and elimination of tobacco-related disparities is listed as one of the four milestones for comprehensive tobacco control^7^.

Although a number of studies have identified smoking trajectories and assessed the underlying factors^8–29^, lifelong designs have been scarce. Smoking is commonly initiated in adolescence or early adulthood^4,30^, and seems to associate with individual and family characteristics in early life^29^, which is why most previous studies have focused on these periods. However, more comprehensive approaches extending over the entire life course are necessary in order to detect smoking patterns also in later life. Lifelong designs seem relevant as several previous reports have concluded that the duration of smoking (i.e., the number of years smoked over the life course) predicts the risk of chronic obstructive pulmonary disease and several smoking-related cancers more accurately than the intensity of smoking (i.e., the average number of cigarettes smoked per day) or pack-years (i.e., duration multiplied by intensity)^31–35^. Recent evidence has associated even low-intensity smoking with increased all-cause mortality in the long term^36^, indicating that accurate characterization of a population’s lifelong smoking patterns is likely to benefit the assessment of subsequent risk of comorbidity and mortality.

In this study, we aim to provide a detailed characterization of the lifelong smoking trajectories of a large unselected Northern Finnish birth cohort population. Using extensive data on the study population’s smoking behaviour, we first profile each individual’s smoking history from the age of 5 to 47 in a year-by-year manner and conduct a latent class trajectory analysis on the population data. We then characterize the identified smoking trajectory classes in terms of sociodemographic and lifestyle variables collected at three time points over the lifelong follow-up of the cohort. Lastly, we perform a longitudinal multivariable comparison between the heaviest smokers and less intense smokers, in an attempt to reveal specific sociodemographic and lifestyle factors that predict non-smoking and discontinued smoking. We expect future initiatives to benefit from our data by exploiting the identified predictors as direct targets of intervention, or as a means of identifying individuals who may benefit from such interventions.

## Methods

### Study sample

**Figure 1:**
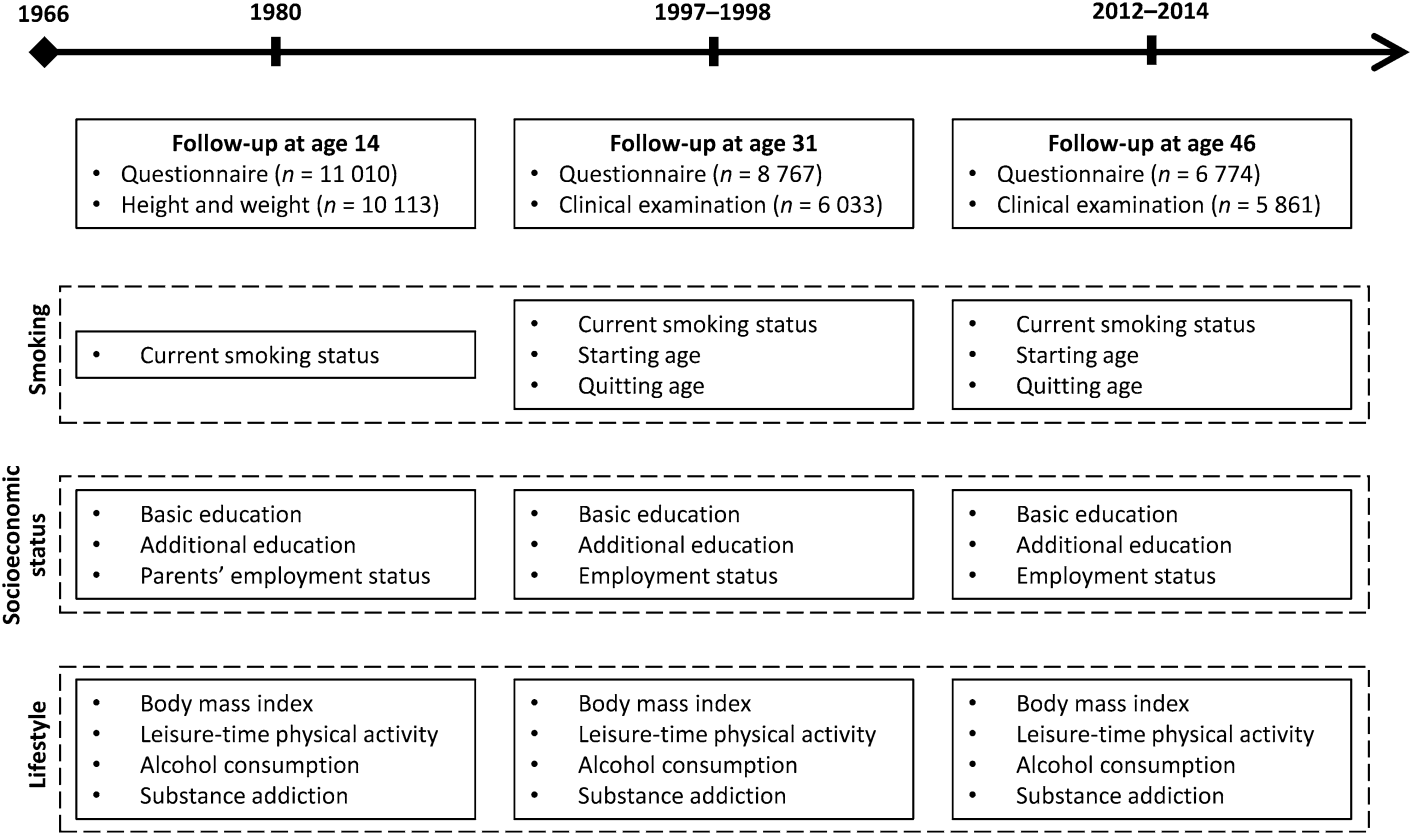
Timeline of the study.

The material of this study stems from the prospective population-based Northern Finland Birth Cohort 1966 (NFBC1966) study^37^. In 1965–1966, pregnant women who resided in Northern Finland (provinces of Oulu and Lapland) with expected dates of delivery during 1966 were recruited into the cohort. Initially, the study covered 12 231 children, corresponding to 96.3% of all births in the area at the time. Major follow-ups took place in 1980, 1997–1998 and 2012–2014, when the NFBC1966 members were 14, 31 and 46 years old, respectively. Figure 1 provides a timeline of the follow-ups and a summary of the data used in the present study. The present sample was comprised of individuals who had responded to the smoking questionnaires at all three follow-ups (n = 5797). Exclusions were solely based on missing data.

### Smoking behaviour

The NFBC1966 cohort members self-reported their tobacco smoking behaviour at several time points during the lifelong follow-up of the cohort (Fig. 1). In the 31- and 46-year follow-up questionnaires, all participants were asked whether they had ever smoked tobacco during their lives. Tobacco was defined as filter cigarettes, other cigarettes, pipes and cigars. The individuals who responded positively were asked to report the age(s) at which they had started smoking (i.e., starting age) and, in a subsequent question, also the age(s) at which they had possibly quit (i.e., quitting age). Starting and quitting ages were reported to the accuracy of one year. For ages of ≤ 31, the values reported in the 31-year questionnaire were the primary source of data. For ages of > 31, or if the 31-year data were missing, we used the 46-year values. Based on the starting and quitting ages, we created 43 binary variables reflecting the annual smoking status of each individual (smoker/non-smoker) from the age of 5 to 47. At each time point *t*, an individual was considered a ’smoker’ if the following conditions were fulfilled: age at initiation ≤ *t*, and age at cessation ≥ *t* or missing.

### Sociodemographic and lifestyle characteristics

As part of the follow-ups at the ages of 14, 31 and 46, the NFBC1966 members were asked about several background characteristics regarding their sociodemographic status and lifestyle. Figure 1 summarizes these data.

#### Education

Basic and additional education were elicited at the ages of 31 and 46. We took into account the most recent data on each individual. Basic education (which includes the Finnish compulsory education of nine years and an optional three years of high school leading to the matriculation examination) was reported using the following options: ‘(1) Less than nine years of compulsory school, (2) Compulsory school, (3) Completion of matriculation examination.’ Additional education (defined as the highest level of other education completed) was reported from the following choices: ‘(1) None, (2) Occupational course, (3) Vocational school, (4) Other lower-level institute/academy/college, (5) Polytechnic, (6) University, (7) Other, (8) Not yet completed.’ An individual was considered to have ‘low education’ if they had not completed secondary or tertiary education (i.e., matriculation examination, vocational school, polytechnic, or university).

#### Employment

Participants self-reported their parents’ employment status at the age of 14 and own employment status at the ages of 31 and 46. At the age of 14, parental employment was reported by completing two statements: ‘My mother is… (1) Staying at home (housewife), (2) Working outside of home, (3) Currently unemployed, (4) On a sick leave, (5) Retired’ and ‘My father is… (1) Working, (2) Unemployed, (3) On a sick leave, (4) Retired.’ The participants were also asked whether they lived with their mother and/or father. If the participant lived in a family with no employed parent(s) (including stay-at-home mothers and permanently retired parents), the family was considered ‘unemployed’.

At the ages of 31 and 46, the participants were asked to respond to the question: ‘Which of the following describes your current employment status best?’ The response options at the age of 31 were: ‘(1) Permanent full-time employee, (2) Temporary full-time employee, (3) Part-time employee, (4) Self-employed, (5) Entrepreneur, (6) Full-time student, (7) Unemployed, (8) Employed/educated through labour market support, (9) Laid off or reduced working hours, (10) Maternity/paternity leave or parental leave, (11) Retired, (12) Other.’ The response options at the age of 46 were: ‘(1) Permanent full-time employee, (2) Permanent part-time employee, (3) Temporary full-time employee, (4) Temporary part-time employee, (5) Full-time self-employed or entrepreneur, (6) Part-time self-employed or entrepreneur, (7) Full-time student, (8) Part-time student, (9) Unemployed for < 6 months, (10) Unemployed for 6–12 months, (11) Unemployed for > 12 months, (12) Employed/educated through labour market support, (13) Laid off or reduced working hours, (14) Maternity/paternity leave or parental leave, (15) Retired, (16) Caring for my own household, (17) Other.’ At both time points, an individual was considered ‘unemployed’ if they were not working or studying full-time or part-time, self-employed or entrepreneur, employed/educated by labour market support, or on parental leave.

#### Obesity

Participants underwent objective height and weight measurements according to previously described methods as part of routine growth monitoring in childhood and adolescence, and as part of the clinical examinations at the ages of 31 and 46^38^. At each follow-up point, body mass index (BMI, kg/m^2^) was calculated as weight (kg) divided by height (m) squared. In accordance with the definitions of WHO, ‘obesity’ was defined as a crude BMI of > 26.5 kg/m^2^ among 14-year-old boys, > 27.8 kg/m^2^ among 14-year-old girls, and ≥ 30 kg/m^2^ among adult men and women^39,40^. For 14-year-olds, we used the BMI cut-offs for individuals aged 14.5 years as the data collections were organized over a longer period of time during the year.

#### Physical activity

Participants self-reported leisure-time physical activity at the ages of 14, 31 and 46 by responding to the following questions. At the age of 14: ‘How often do you participate in sports outside school hours? (1) Daily, (2) Every other day, (3) Twice a week, (4) Once a week, (5) Every other week, (6) Once a month, (7) Generally not at all.’ At the ages of 31 and 46: ‘How often do you participate in brisk physical activity/exercise [defined as causing at least some sweating and breathlessness] during your leisure time? (1) Daily, (2) 4–6 times a week, (3) 2–3 times a week, (4) Once a week, (5) 2–3 times a month, (6) Once a month or less often.’ At each time point, an individual was considered ‘inactive’ if they participated in sports/physical activity less than once a week.

#### Alcohol consumption

Participants self-reported alcohol consumption at the ages of 14, 31 and 46 by responding to the following questions. At the age of 14: ‘Do you consume alcohol [defined as beer or any other drink containing alcohol]? (1) Never, (2) Experimented once, (3) Experimented a few times, (4) Yes, on a monthly basis, (5) Yes, on a weekly basis.’ At the ages of 31 and 46: ‘Do you currently consume any alcoholic beverages (e.g., beer, cider, low-alcohol wines, wine or spirits) even occasionally? (1) I have never consumed alcohol, (2) No, I have quit drinking, (3) Yes, less than once a month, (4) Yes, at least once a month.’ At each time point, an individual was considered a ‘regular drinker’ if they consumed alcohol at least once a week.

#### Substance addiction

Participants self-reported substance-related behaviour (defined as other than alcohol or tobacco) at the ages of 14, 31 and 46. At the age of 14, the participants were asked to complete the statement: ‘Other substances… (1) Never experimented, (2) Experimented once, (3) Experimented several times, (4) I use regularly.’ Regular users were considered to have ‘substance addiction’. At the age of 31 and 46, the questionnaires elicited directly whether the participant had a ‘substance addiction’ (yes/no).

### Statistical analysis

#### Modelling of lifelong smoking behaviour

We profiled the lifelong smoking behaviour of the sample by means of latent class growth modelling (LCGM). LCGM is a semi-parametric statistical modelling method that aims to reveal latent groups of individuals (i.e., classes) following a distinct pattern of change (i.e., trajectory) over time^41^. The LCGM analysis was performed using the SAS version 9.4 (SAS Institute Inc., Cary, NC, USA), the PROC TRAJ macro and the logistic LOGIT model for binary data^42^. First, models with one to six classes were fitted to the data, after which the most adequate model was chosen according to the following measures of model adequacy: (1) Bayesian information criterion and Akaike information criterion (BIC and AIC, respectively; lower values indicate better fit); (2) Posterior membership probability (averages should exceed 0.70); (3) Absolute and relative class sizes, also taking into consideration the subsequent analyses; and (4) Clinical significance of the models^41^. Once the most suitable model was selected, the participants were classed according to the highest posterior membership probabilities^41^. As the smoking trajectories of men and women were highly similar in our data (data not shown), both sexes were modelled together in order to obtain equivalent definitions of each smoking trajectory among men and women. This approach was also supported by the findings of a previous study, which detected no difference in the latent class structure between the multi-decadal smoking trajectory models (age 18 to 50) of men and women^26^.

#### Profiles of smoking trajectory classes and between-class comparisons

In order to further describe the identified smoking trajectory classes, we presented the distributions of all background variables for each class separately. As the variables were dichotomous, frequencies and percentages were presented.

To address the associations between background variables and smoking trajectory class in a longitudinal, multivariable analysis, we used a generalized estimating equations (GEE) approach. An extension to regression-based methods, GEE is able to correct for correlations within data (such as temporal dependencies due to repeated measurements) by means of a working correlation matrix^43,44^. Here, we used the binary logistic main-effects GEE model with ‘exchangeable’ working correlation matrix structure. Each background variable had their own model, with smoking trajectory class as the main predictor and the other background variables as covariates. Sex, education, and smoking trajectory class were fixed (i.e., time-invariant) variables, whereas all the other variables were considered to be repeated measurements (i.e., records from three time points) and thus nested within individuals. As we aimed to reveal specific factors which predict non-smoking and discontinued smoking, we chose the heaviest smokers’ class to be compared with the other smoking trajectory classes. Exponentiated regression coefficients (i.e., odds ratios, ORs), their 95% Wald confidence intervals (CIs) and the corresponding P values were documented from the data output.

#### Analysis of representativeness

To address the potential selection bias associated with the long follow-up and consequent attrition, we studied the differences between the present smoking trajectory sample and the rest of the NFBC1966 population. The background variables were compared between the sample and those excluded by means of Chi square tests.

Except for the trajectory modelling, we conducted all the statistical analyses using SPSS version 26 (IBM, Armonk, NY, USA). The threshold for statistical significance was set at *P* = 0.05.

### Ethical considerations

The data were pseudonymized by the NFBC1966 data experts prior to analysis. Informed consent was collected at each stage from the participants and/or their legal guardians. The Declaration of Helsinki was followed, and ethical approvals were obtained from the Ethics Committee of the Northern Ostrobothnia Hospital District (12/2003; 94/2011). The datasets generated and analyzed during the current study are not publicly available due to local privacy regulations but are available from the NFBC Project Center for researchers who meet the criteria for accessing confidential data.

## Results

### Study sample

**Figure 2:**
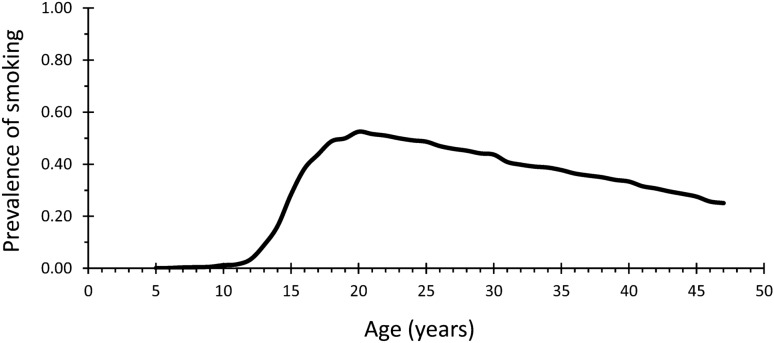
Overall prevalence of smoking from age 5 to 47 in the study population (n = 5797). Supplementary Table 2 presents the annual smoking prevalences in numerical format.

The study sample consisted of 5797 individuals, of whom 44.0% were men and 56.0% women. The full study population’s annual prevalence of smoking from the age of 5 to 47 is presented in Fig. 2. After the age of 10, the prevalence steeply increased, reaching its maximum of 52.5% at the age of 20, with a mild but steady decrease thereafter. The cumulative prevalence of smoking (i.e., percentage of ever-smokers) in the sample was 60.8%. Supplementary Table 1 shows the comparison of the present sample to those excluded. The drop-outs were characterized by varyingly higher rates of male sex, low education, unemployment, obesity, physical inactivity, regular drinking, and substance addiction than the present sample.

**Table 1:** Fit statistics from trajectory models with one to six classes.

Number of classes	|BIC|	|AIC|	Relative class sizes (%)	Posterior membership probabilities (class means)
1	137,860.4	137,845.8	100	1.00
2	62,163.4	62,140.0	61.2/38.8	1.00/0.99
3	46,184.9	46,148.2	47.7/18.4/33.9	1.00/0.99/1.00
4	40,553.1	40,503.1	43.7/29.4/16.6/10.3	1.00/1.00/0.99/0.98
5	38,970.2	38,910.2	16.0/45.3/13.5/20.7/4.4	0.99/1.00/1.00/0.99/0.98
6	35,259.0	35,189.0	10.8/12.6/10.5/20.7/4.3/41.0	0.99/0.98/1.00/0.99/0.99/1.00

*AIC* Akaike information criterion, *BIC* Bayesian information criterion.

### Smoking trajectory analysis

**Figure 3:**
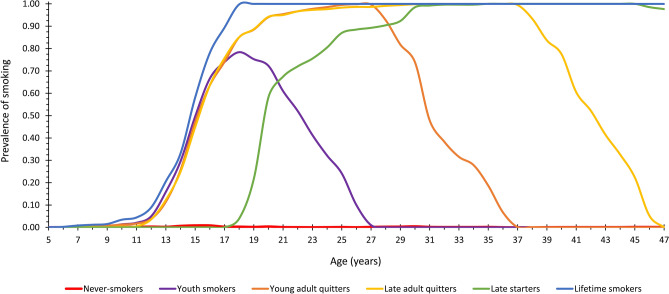
Six distinct trajectories for lifelong smoking behaviour among the study population (n = 5797). Supplementary Table 2 presents the annual smoking prevalences of each class in numerical format.

Of the LCGM models with one to six smoking trajectories (fit statistics listed in Table 1), we selected the six-class model as the most adequate. It had considerably lower BIC and AIC values than the corresponding models with one to five groups, and importantly, was the only model to make a distinction between never-smokers and youth smokers. Figure 3 presents the six identified smoking trajectories. Each of the trajectories was considered to represent a clear, distinct pattern in terms of lifelong smoking behaviour, and the corresponding classes were subsequently named as follows: never-smokers (relative class size 41.0% of the sample, n = 2376), youth smokers (12.6%, n = 730), young adult quitters (10.8%, n = 627), late adult quitters (10.5%, n = 611), late starters (4.3%, n = 252) and lifetime smokers (20.7%, n = 1201).

### Profiles of smoking trajectory classes

Background characteristics of the smoking trajectory classes are presented in Table 2. Generally, the smokers’ classes tended to include more individuals who were male, had low education level, were unemployed, obese, physically inactive and regular drinkers. Lifetime smokers and late adult quitters (i.e., long-term smokers) showed the highest contrast to never-smokers.

**Table 2:** Sociodemographic and lifestyle characteristics among the smoking trajectory classes.

Characteristic	All (n = 5797)	Smoking trajectory class					
		Never-smokers (n = 2376)	Youth smokers (n = 730)	Young adult quitters (n = 627)	Late adult quitters (n = 611)	Late starters (n = 252)	Lifetime smokers (n = 1201)
Male sex	44.0 (2552)	39.2 (932)	38.8 (283)	52.0 (326)	54.5 (333)	51.2 (129)	45.7 (549)
Low education^1^	22.2 (1277)	18.9 (447)	24.2 (176)	22.5 (140)	24.7 (150)	24.6 (62)	25.3 (302)
**Unemployment**^**2**^							
At age 14	13.4 (776)	13.1 (311)	14.1 (103)	12.6 (79)	13.6 (83)	9.9 (25)	14.6 (175)
At age 31	13.4 (768)	11.0 (259)	13.7 (99)	12.7 (78)	15.1 (92)	13.3 (33)	17.4 (207)
At age 46	11.9 (668)	9.7 (225)	10.7 (76)	10.8 (66)	11.3 (67)	16.0 (39)	16.9 (195)
**Obesity**^**3**^							
At age 14	1.1 (57)	0.7 (15)	0.7 (5)	1.9 (11)	1.4 (8)	2.2 (5)	1.2 (13)
At age 31	8.1 (346)	5.6 (132)	6.7 (36)	8.3 (38)	9.1 (42)	10.8 (20)	8.7 (78)
At age 46	20.3 (974)	15.7 (373)	18.9 (118)	21.8 (116)	26.0 (129)	22.8 (44)	21.5 (194)
**Physical inactivity**^**4**^							
At age 14	23.9 (1368)	21.2 (498)	25.4 (184)	23.4 (147)	24.9 (150)	21.4 (53)	28.4 (336)
At age 31	32.3 (1873)	28.2 (666)	29.9 (217)	33.4 (209)	36.8 (224)	29.9 (75)	40.3 (482)
At age 46	27.2 (1554)	22.9 (539)	23.8 (171)	27.6 (173)	28.5 (170)	30.0 (75)	36.0 (426)
**Regular drinking**^**5**^							
At age 14	0.5 (31)	0.1 (3)	0.7 (5)	1.8 (11)	0.5 (3)	0.0 (0)	0.8 (9)
At age 31	42.3 (2435)	36.2 (860)	42.1 (305)	47.0 (293)	46.8 (285)	49.6 (124)	47.7 (568)
At age 46	52.7 (3039)	45.2 (1075)	54.9 (401)	57.8 (361)	58.2 (353)	58.2 (146)	58.9 (703)
**Substance addiction**^**6**^							
At age 14	0.0 (0)	0.0 (0)	0.0 (0)	0.0 (0)	0.0 (0)	0.0 (0)	0.0 (0)
At age 31	0.2 (10)	0.0 (1)	0.0 (0)	0.3 (2)	0.3 (2)	0.4 (1)	0.3 (4)
At age 46	0.5 (28)	0.2 (5)	0.1 (1)	0.6 (4)	0.7 (4)	0.8 (2)	1.0 (12)

Values are presented as: Percentage (frequency). N varies due to missing sociodemographic/lifestyle data.

^1^Primary education only.

^2^Parental unemployment at age 14; own unemployment at age 31 and 46.

^3^According to body mass index, following definitions of World Health Organization.

^4^Leisure-time physical activity < 1/week.

^5^Drinking ≥ 1/week.

^6^Regular substance use at age 14; self-reported substance addiction at age 31 and 46.

### Comparison of lifetime smokers to other smoking trajectory classes

To reveal specific sociodemographic and lifestyle factors that discriminate between heavy smokers and less intense smokers, we compared the heaviest smokers’ class (i.e., lifetime smokers) to the other smoking trajectory classes. The corresponding results from multivariable GEE models are presented in Table 3. There was an obvious distinction between lifetime smokers and never-smokers in the GEE models, as male sex, low education, unemployment, physical inactivity and regular drinking were each independently and significantly associated with increased odds of belonging to the lifetime smokers’ class. Youth smoking was associated with female sex, employment and physical activity. Successful adult quitting was associated with male sex and obesity, whereas unemployed and physically inactive had higher odds of belonging to the lifetime smokers’ class. Late starting was associated with employment and physical activity.

**Table 3:** Multivariable generalized estimating equations (GEE) analysis addressing the association of sociodemographic and lifestyle characteristics with smoking trajectory.

Characteristic	Lifetime smokers compared to…														
	Never-smokers			Youth smokers			Young adult quitters			Late adult quitters			Late starters		
	OR	95% CI	P	OR	95% CI	P	OR	95% CI	P	OR	95% CI	P	OR	95% CI	P
Male sex	**1.29**	**1.12; 1.49**	** < 0.001**	**1.33**	**1.10; 1.61**	**0.003**	**0.77**	**0.64; 0.94**	**0.010**	**0.71**	**0.58; 0.86**	**0.001**	0.82	0.62; 1.07	0.144
Low education	**1.44**	**1.22; 1.71**	** < 0.001**	1.06	0.85; 1.31	0.621	1.16	0.92; 1.46	0.213	1.03	0.82; 1.30	0.788	1.03	0.75; 1.42	0.853
Unemployment	**1.45**	**1.26; 1.66**	** < 0.001**	**1.31**	**1.08; 1.57**	**0.005**	**1.38**	**1.14; 1.68**	**0.001**	**1.28**	**1.06; 1.55**	**0.009**	**1.38**	**1.04; 1.82**	**0.025**
Obesity	1.04	0.86; 1.24	0.712	1.08	0.85; 1.37	0.540	0.88	0.69; 1.12	0.298	**0.79**	**0.63; 0.99**	**0.047**	0.78	0.56; 1.10	0.158
Physical inactivity	**1.52**	**1.35; 1.70**	** < 0.001**	**1.41**	**1.22; 1.64**	** < 0.001**	**1.27**	**1.10; 1.48**	**0.002**	**1.20**	**1.03; 1.40**	**0.016**	**1.37**	**1.10; 1.71**	**0.005**
Regular drinking	**1.32**	**1.19; 1.46**	** < 0.001**	0.99	0.87; 1.12	0.831	0.91	0.80; 1.04	0.186	1.04	0.91; 1.20	0.535	1.02	0.85; 1.23	0.831
Substance addiction	1.98	0.68; 5.76	0.212	3.76	0.46; 30.6	0.216	0.66	0.21; 2.09	0.481	0.98	0.29; 3.29	0.969	0.80	0.17; 3.74	0.778

Odds ratios (OR) with 95% confidence intervals (CI) from logistic GEE models. Lifetime smokers are compared to the other smoking trajectories, i.e., OR > 1 indicates increased odds of belonging to the lifetime smokers’ trajectory, and OR < 1 indicates increased odds of belonging to the reference trajectory.

Bold denotes statistical significance.

## Discussion

Among 5797 Northern Finns followed up for 46 years, this population-based birth cohort study identified six distinct trajectories for smoking behaviour across the life course: never-smokers, youth smokers, young adult quitters, late adult quitters, late starters, and lifetime smokers. Generally, the smokers’ classes tended to include more individuals who were male, had lower socioeconomic status and unhealthier lifestyle. Multivariable comparisons between lifetime smokers and the other smoking trajectory classes identified unemployment and physical inactivity as significant predictors of lifetime smoking relative to any other class. Female sex increased the odds of never-smoking and youth smoking, whereas male sex increased the odds of adult quitting.

According to worldwide predictions, over one billion individuals will remain smokers in 2025 if current smoking trends remain constant^6^, leading to nearly 500 million smoking-related deaths between 2000 and 2050^45^. Although the Nordic countries have a relatively low prevalence of smoking on the global scale^4^, it has been estimated that up to 15% of health care expenditure in high-income countries is attributed to smoking^46^. Thus, it is clear that zero smoking is the ideal for both the individual and society^1^. In 2018, 15% of Finnish working-aged men and 13% of women were current smokers, respectively; the prevalence figures have clearly decreased over time^47^. However, the present study showed that 60% of the now middle-aged Northern Finnish population had smoked at some point in their lives, and that 25% remained smokers at midlife, emphasizing the need for a detailed characterization of smoking behaviour and underlying factors specifically among this population. In this study, we were able to exploit an explicit dataset that enabled the assessment of annual smoking status of each individual from the age of 5 to 47.

The present analysis identified six trajectories for lifelong smoking behaviour. One of these represented never-smokers, two current smokers (lifetime smokers and late starters), and three ex-smokers (youth smokers, young adult quitters, and late adult quitters). As reflected in their names, each of the six trajectories represented a clear, distinct smoking pattern. Each trajectory also showed a reasonable class size and high average posterior membership probability, indicating that the final LCGM model was robust and the identified trajectories were truthful. Most previous studies have also described similar trajectories, depending on the study population and age period modelled^8–29^; for example, studies focusing on adolescence and early adulthood have commonly presented more detailed trajectories regarding the initiation of smoking but have correspondingly lacked trajectories representing those who quit in later life.

The present analyses regarding the association of smoking trajectory with sociodemographic and lifestyle characteristics give ground for several remarks. Firstly, our data confirm the previously known coexistence of low socioeconomic status, unhealthy lifestyle, and smoking; our study setting enabled us to demonstrate this in a longitudinal manner from adolescence to midlife. Secondly, our multivariable GEE models which compared lifetime smokers with other smoking trajectory classes revealed statistically significant differences between classes. Youth smoking (i.e., smoking limited to late adolescence and early adulthood) was associated with female sex, employment and physical activity. Young and late adult quitting (i.e., successful quitting in adulthood) were positively associated with male sex and negatively associated with unemployment and physical inactivity. Late starting (i.e., starting in adulthood) was associated with employment and physical activity.

In general, the differences have been observed in previous studies (e.g., association of smoking with sex^10,11,14^, low education^14,27^, unemployment^27^, low socioeconomic status^23^, and alcohol or substance use^9,28^), though mostly among adolescents, young adults, or non-general population samples. Moreover, most previous studies have only been able to assess a limited number of background variables, without a longitudinal multivariable approach concerning both smoking behaviour and background variables. In our lifelong approach, the disparities in sociodemographic and lifestyle characteristics between smoking trajectory classes could be observed in a longitudinal manner, i.e., from adolescence to midlife, indicating that they provide means for early detection of individuals at higher risk of starting and continuing smoking. For each smoking trajectory class, we were able to present at least two sociodemographic or lifestyle-related characteristics that statistically differentiate the class from lifetime smokers.

The identified characteristics should serve as primary targets for future interventions and preventive/supportive measures. First, our data highlight the importance of interventions aimed at adolescents aged 10 to 20 years; in four out of five ever-smokers trajectories, the prevalence of smoking exceeded 50% by the age of 16. Young adolescents should be informed (e.g., by parents, teachers, school nurses and sports coaches) about nicotine dependence and the health effects of short-term and long-term smoking, and older adolescents should also be offered cessation support. Second, unemployed individuals constitute a clear target group for interventions, regardless of age. Unemployment security services should actively promote cessation support, and health checks of the unemployed should routinely include enquiry about smoking history and open discussion regarding the health effects of smoking. Third, physical inactivity was associated with lifetime smoking. Inactivity is typically coupled with increased sedentary time, implying that electronic (e.g., mobile application-, internet-, or television-mediated) interventions may prove effective among these individuals. We expect future initiatives to benefit from our data by exploiting the identified predictors as direct targets of intervention, or as a means of identifying individuals who may benefit from such interventions.

The main strength of this study was its long follow-up period, extending over the life course of the NFBC1966 population. As a population-based birth cohort, the NFBC1966 provided the best available estimate of the general population, with extensive data on smoking behaviour, sociodemographics and lifestyle across the life course. Despite the long follow-up, the sample size remained large (n = 5797), favouring the population-based setting of the study. Importantly, the smoking trajectories were based on annual data on smoking from the age of 5 to 47, which enhanced the accuracy of trajectory estimation. A lifelong approach was considered highly valuable in order to detect the potential trajectories that represent a change in smoking behaviour in later life.

There were also limitations to our study. First, it was based on self-reported smoking data, which raises the question as to whether our dataset was somewhat affected by social desirability or some other type of response bias. Further, despite the large study sample (n = 5797), a significant number of those originally born into the cohort (52.6%) dropped out at some point during the follow-up. The fact that the cohort study was initiated in 1966 and the follow-up extended over five decades explains the high drop-out to some extent, but also suggests that our data may be subject to selection bias. Our analysis of representativeness confirmed the mild differences between the sample and those excluded, as the drop-outs were characterized by varyingly higher rates of male sex, lower socioeconomic status, and unhealthier lifestyle. While we fully acknowledge that our data are affected by selection bias, we point out that both response bias and selection bias would primarily cause our data to underestimate the prevalence of smoking such that non-smokers would be over-estimated and ever-smokers would be underestimated in the current data. Second, our study setting prevented us from addressing causal relationships between smoking and background variables. As such, we reported mere associations between variables without implying the direction of the association. We also used the term ‘predictor’ in a neutral sense to refer to an independent variable in a statistical model. Third, we did not assess the intensity of smoking, because self-reports of average smoking intensity are typically subject to imprecision^31^, and because previous reports had concluded that the duration of smoking predicts smoking-related comorbidities more accurately than intensity^31–35^. Importantly, growing evidence has also associated even low-intensity smoking with increased mortality in the long-term^36^, further emphasizing the predictive value of smoking duration over intensity. Fourth, this study addressed only tobacco smoking, defined as filter cigarettes, other cigarettes, pipes and cigars. Passive exposure to tobacco smoke, as well as exposure to other tobacco products such as snus and electronic cigarettes, were omitted. In this currently middle-aged study population, the use of new tobacco products seemed to be minor and was considered to have a minimal effect on lifelong smoking patterns. The population’s lifelong exposure to tobacco smoke is likely to be considerable, but we lacked annual data on passive smoking exposure and therefore decided to focus our approach on active smoking.

## Conclusion

In this birth cohort study of 5797 Northern Finns followed up for 46 years, we identified six trajectories for lifelong smoking behaviour: one trajectory representing never-smokers, two representing current smokers, and three representing ex-smokers. Smoking was generally associated with male sex, lower socioeconomic status and unhealthier lifestyle. Detailed between-class comparisons showed that unemployment and physical inactivity were significant predictors of lifetime smoking relative to any other smoking trajectory class. Female sex increased the odds of never-smoking and youth smoking, whereas male sex increased the odds of adult quitting, relative to lifetime smoking. We expect future initiatives to benefit from our data by exploiting the identified predictors as direct targets of intervention, or as a means of identifying individuals who may benefit from such interventions.

## Supplementary information

Supplementary Information.

## Data Availability

The datasets generated and analyzed during the current study are not publicly available due to local privacy regulations but are available from the NFBC Project Center for researchers who meet the criteria for accessing confidential data.

## Abbreviations

AIC: Akaike information criterion
BIC: Bayesian information criterion
CI: Confidence interval
GEE: Generalized estimating equations
LCGM: Latent class growth modelling
NFBC1966: Northern Finland Birth Cohort 1966
OR: Odds ratio
WHO FCTC: World Health Organization’s Framework Convention on Tobacco Control
