# Supplementary material for: Lifelong smoking trajectories of Northern Finns are characterized by sociodemographic and lifestyle differences in a 46-year follow-up
Source: Sci Rep. 2020 Oct 1;10:16365. doi: 10.1038/s41598-020-73334-3 (PMC7529914; doi:10.1038/s41598-020-73334-3)
Supplement: Supplementary file 1 — Supplementary Information. [file 41598_2020_73334_MOESM1_ESM.docx]

TITLE PAGE FOR SUPPLEMENTARY DATA

MANUSCRIPT TITLE
**Lifelong smoking trajectories of Northern Finns are characterized by sociodemographic and lifestyle differences in a 46-year follow-up**

AUTHOR LIST
Petteri Oura^1^, MD, PhD, petteri.oura@oulu.fi

Ina Rissanen^2,3,4^, MD, PhD

Juho-Antti Junno^5^, PhD

Terttu Harju^6^, MD, PhD

Markus Paananen^1^, MD, PhD

AFFILIATIONS AND ADDRESSES

^1^Center for Life Course Health Research, Faculty of Medicine, University of Oulu, Oulu, Finland
PO Box 5000, FI-90014 University of Oulu, Finland

^2^Departments of Neurology and Neurosurgery, Oulu University Hospital, Finland
PO Box 50, FI-90029 OYS, Finland

^3^Medical Research Center Oulu (MRC Oulu), Northern Ostrobothnia Hospital District and University of Oulu, Finland
PO Box 5000, FI-90014 University of Oulu, Finland

^4^Julius Center for Health Sciences and Primary Care, University Medical Center Utrecht, The Netherlands
PO Box 85500, NL-3508 GA Utrecht, The Netherlands

^5^Cancer Research and Translational Medicine Research Unit, University of Oulu, Oulu, Finland
PO Box 5000, FI-90014 University of Oulu, Finland

^6^Research Unit of Internal Medicine, Faculty of Medicine, University of Oulu, Oulu, Finland
PO Box 5000, FI-90014 University of Oulu, Finland

**Supplementary Table 1**. Comparison of individuals included in the sample (n = 5797) to those excluded due to missing data (932 ≤ n ≤ 6336).

| Characteristic | Sample | Excluded | P for difference |
| --- | --- | --- | --- |
| Male sex | 44.0 (2552) | 57.8 (3661) | **<0.001** |
| Low education^1^ | 22.2 (1277) | 26.5 (296) | **0.002** |
| Unemployment^2^ |  |  |  |
| At age 14 | 13.4 (776) | 31.2 (1979) | **<0.001** |
| At age 31 | 13.4 (768) | 20.3 (575) | **<0.001** |
| At age 46 | 11.9 (668) | 19.5 (209) | **<0.001** |
| Obesity^3^ |  |  |  |
| At age 14 | 1.1 (57) | 1.6 (76) | **0.012** |
| At age 31 | 8.1 (346) | 11.0 (188) | **<0.001** |
| At age 46 | 20.3 (974) | 26.5 (271) | **<0.001** |
| Physical inactivity^4^ |  |  |  |
| At age 14 | 23.9 (1368) | 25.2 (1257) | 0.114 |
| At age 31 | 32.3 (1873) | 37.7 (1063) | **<0.001** |
| At age 46 | 27.2 (1554) | 30.8 (305) | **0.018** |
| Regular drinking^5^ |  |  |  |
| At age 14 | 0.5 (31) | 0.3 (14) | **0.036** |
| At age 31 | 42.3 (2435) | 41.8 (1176) | 0.675 |
| At age 46 | 52.7 (3039) | 53.5 (541) | 0.628 |
| Substance addiction^6^ |  |  |  |
| At age 14 | 0.0 (0) | 0.0 (0) | - |
| At age 31 | 0.2 (10) | 0.6 (18) | **<0.001** |
| At age 46 | 0.5 (28) | 1.2 (12) | **0.007** |

Values are presented as: Percentage (frequency). N varies due to missing sociodemographic/lifestyle data.

^1^Primary education only. ^2^Parental unemployment at age 14; own unemployment at age 31 and 46. ^3^According to body mass index, following definitions of World Health Organization. ^4^Leisure-time physical activity <1/week. ^5^Drinking ≥1/week. ^6^Regular substance use at age 14; self-reported substance addiction at age 31 and 46.

**Supplementary Table 2**. Prevalence of smoking from ages 5 to 47 among the full study population and each smoking class. **Figure 2** (all) and **Figure 3** (by class) present the data in graphical format.

| Age (years) |  | Prevalence of smoking (%) | | | | | | | |
| --- | --- | --- | --- | --- | --- | --- | --- | --- | --- |
|  |  | All |  | By class | | | | | |
|  |  |  |  | Never-smokers | Youth smokers | Young adult quitters | Late adult quitters | Late starters | Lifetime smokers |
| 5 |  | 0.0 |  | 0.0 | 0.1 | 0.0 | 0.0 | 0.0 | 0.0 |
| 6 |  | 0.1 |  | 0.1 | 0.1 | 0.0 | 0.0 | 0.0 | 0.2 |
| 7 |  | 0.3 |  | 0.1 | 0.4 | 0.3 | 0.2 | 0.0 | 0.8 |
| 8 |  | 0.4 |  | 0.1 | 0.4 | 0.6 | 0.2 | 0.0 | 1.2 |
| 9 |  | 0.6 |  | 0.2 | 0.5 | 0.8 | 0.2 | 0.0 | 1.5 |
| 10 |  | 1.2 |  | 0.3 | 1.4 | 1.3 | 0.3 | 0.0 | 3.4 |
| 11 |  | 1.5 |  | 0.2 | 2.1 | 1.8 | 0.5 | 0.0 | 4.4 |
| 12 |  | 3.4 |  | 0.3 | 4.9 | 3.8 | 3.6 | 0.0 | 8.9 |
| 13 |  | 8.9 |  | 0.3 | 15.8 | 11.5 | 12.3 | 0.0 | 20.5 |
| 14 |  | 16.2 |  | 0.8 | 29.0 | 25.7 | 24.9 | 0.0 | 32.9 |
| 15 |  | 28.4 |  | 0.8 | 49.5 | 47.0 | 44.8 | 0.0 | 57.8 |
| 16 |  | 38.3 |  | 0.6 | 67.0 | 63.3 | 63.5 | 0.0 | 77.6 |
| 17 |  | 43.7 |  | 0.1 | 74.1 | 73.8 | 75.6 | 0.0 | 88.8 |
| 18 |  | 48.8 |  | 0.2 | 78.2 | 84.8 | 84.9 | 0.0 | 100.0 |
| 19 |  | 49.9 |  | 0.1 | 74.9 | 88.4 | 88.5 | 19.4 | 100.0 |
| 20 |  | 52.5 |  | 0.3 | 71.6 | 94.1 | 94.1 | 57.1 | 100.0 |
| 21 |  | 51.6 |  | 0.1 | 60.4 | 95.4 | 94.9 | 66.7 | 100.0 |
| 22 |  | 51.0 |  | 0.0 | 51.5 | 96.7 | 96.6 | 71.4 | 100.0 |
| 23 |  | 49.9 |  | 0.0 | 40.7 | 97.8 | 97.2 | 75.0 | 100.0 |
| 24 |  | 49.1 |  | 0.1 | 31.5 | 98.6 | 97.5 | 80.2 | 100.0 |
| 25 |  | 48.6 |  | 0.1 | 23.6 | 99.5 | 98.4 | 86.5 | 100.0 |
| 26 |  | 47.0 |  | 0.0 | 9.5 | 99.8 | 98.7 | 88.1 | 100.0 |
| 27 |  | 45.9 |  | 0.2 | 0.3 | 99.8 | 98.7 | 88.9 | 100.0 |
| 28 |  | 45.2 |  | 0.3 | 0.0 | 92.2 | 99.2 | 90.1 | 100.0 |
| 29 |  | 44.1 |  | 0.3 | 0.0 | 81.3 | 99.5 | 92.1 | 100.0 |
| 30 |  | 43.7 |  | 0.5 | 0.0 | 73.5 | 99.8 | 98.4 | 100.0 |
| 31 |  | 40.8 |  | 0.2 | 0.0 | 47.8 | 100.0 | 99.2 | 100.0 |
| 32 |  | 39.8 |  | 0.1 | 0.0 | 38.4 | 100.0 | 99.6 | 100.0 |
| 33 |  | 39.1 |  | 0.1 | 0.0 | 31.7 | 100.0 | 99.6 | 100.0 |
| 34 |  | 38.7 |  | 0.1 | 0.0 | 28.1 | 100.0 | 99.6 | 100.0 |
| 35 |  | 37.7 |  | 0.2 | 0.0 | 19.0 | 100.0 | 100.0 | 100.0 |
| 36 |  | 36.4 |  | 0.0 | 0.0 | 7.2 | 100.0 | 100.0 | 100.0 |
| 37 |  | 35.6 |  | 0.0 | 0.0 | 0.2 | 100.0 | 100.0 | 100.0 |
| 38 |  | 35.0 |  | 0.0 | 0.0 | 0.2 | 93.8 | 100.0 | 100.0 |
| 39 |  | 33.9 |  | 0.0 | 0.0 | 0.0 | 84.1 | 100.0 | 100.0 |
| 40 |  | 33.3 |  | 0.1 | 0.0 | 0.0 | 77.9 | 100.0 | 100.0 |
| 41 |  | 31.5 |  | 0.1 | 0.0 | 0.0 | 60.9 | 100.0 | 100.0 |
| 42 |  | 30.6 |  | 0.1 | 0.0 | 0.0 | 52.5 | 100.0 | 100.0 |
| 43 |  | 29.5 |  | 0.1 | 0.0 | 0.0 | 41.7 | 100.0 | 100.0 |
| 44 |  | 28.6 |  | 0.1 | 0.0 | 0.0 | 32.9 | 100.0 | 100.0 |
| 45 |  | 27.5 |  | 0.2 | 0.0 | 0.0 | 22.6 | 100.0 | 100.0 |
| 46 |  | 25.6 |  | 0.2 | 0.0 | 0.0 | 5.2 | 98.8 | 100.0 |
| 47 |  | 25.0 |  | 0.1 | 0.0 | 0.0 | 0.0 | 98.0 | 100.0 |
